# Supplementary material for: Using Household Dietary Diversity Score and Spatial Analysis to Inform Food Governance in Chile
Source: Nutrients. 2024 Sep 2;16(17):2937. doi: 10.3390/nu16172937 (PMC11397145; doi:10.3390/nu16172937)
Supplement: Supplementary file 1 [file nutrients-16-02937-s001.zip › nutrients-3186085-supplementary.pdf]

Table S1 - Intake of different food groups in Chilean vulnerable households.

Number of households								
**Food group**	**Below recommended**	**Recommended**	**Above recommended**	**Weekly intake**				
				**Never**	**Sometimes**	**1-2/week**	**3-5/week**	**Everyday**
Vegetables	2,111	1,936	-	2	130	604	1,226	2,085
Fruit	2,658	1,389	-	14	354	941	1,289	1,449
Dairy	2,639	1,408	-	101	538	860	1,078	1,470
Legumes	764	3,282	1	57	398	2,879	663	50
White meat	683	3,302	62	20	623	2,050	1,291	63
Red meat	1,237	2,272	538	117	1,136	2,245	468	81
Eggs	456	3,038	553	42	406	1,292	1,727	580
Water	572	3,475	-	19	33	70	156	3,769
Cereals	350	3,690	7	3	61	685	1,103	2,195
Bread	321	3,724	2	4	21	51	111	3,860
Fat	253	2,147	1,647	349	1,928	926	445	399
Sugar	248	2,091	1,708	386	1,814	783	446	618

Figure S - Communes with more than 50% of households below/above recommended for each food group across the different Chilean Macro-zone.

1. Figure S1. Fruits
    a. Norte Grande

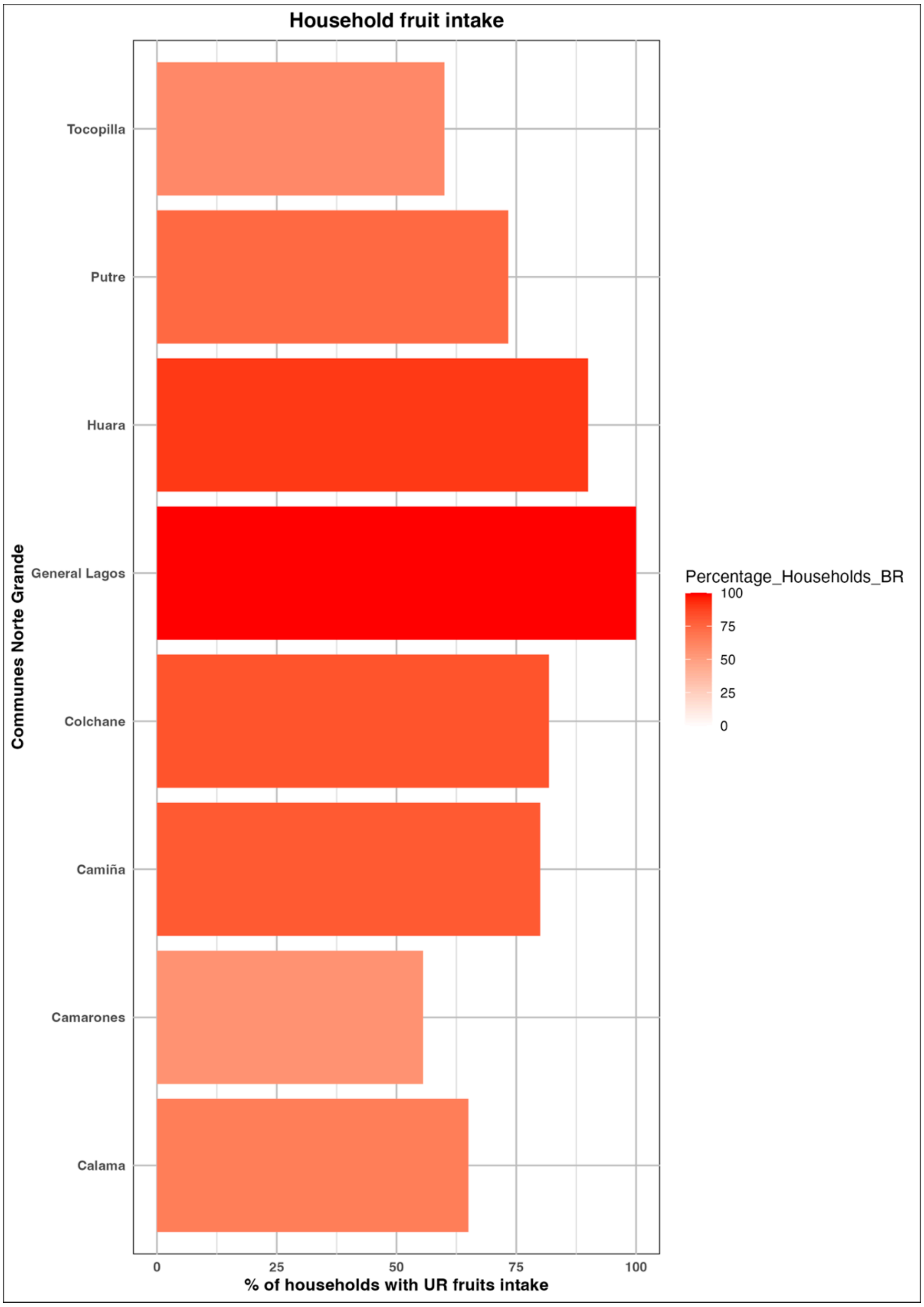

b. Norte Chico

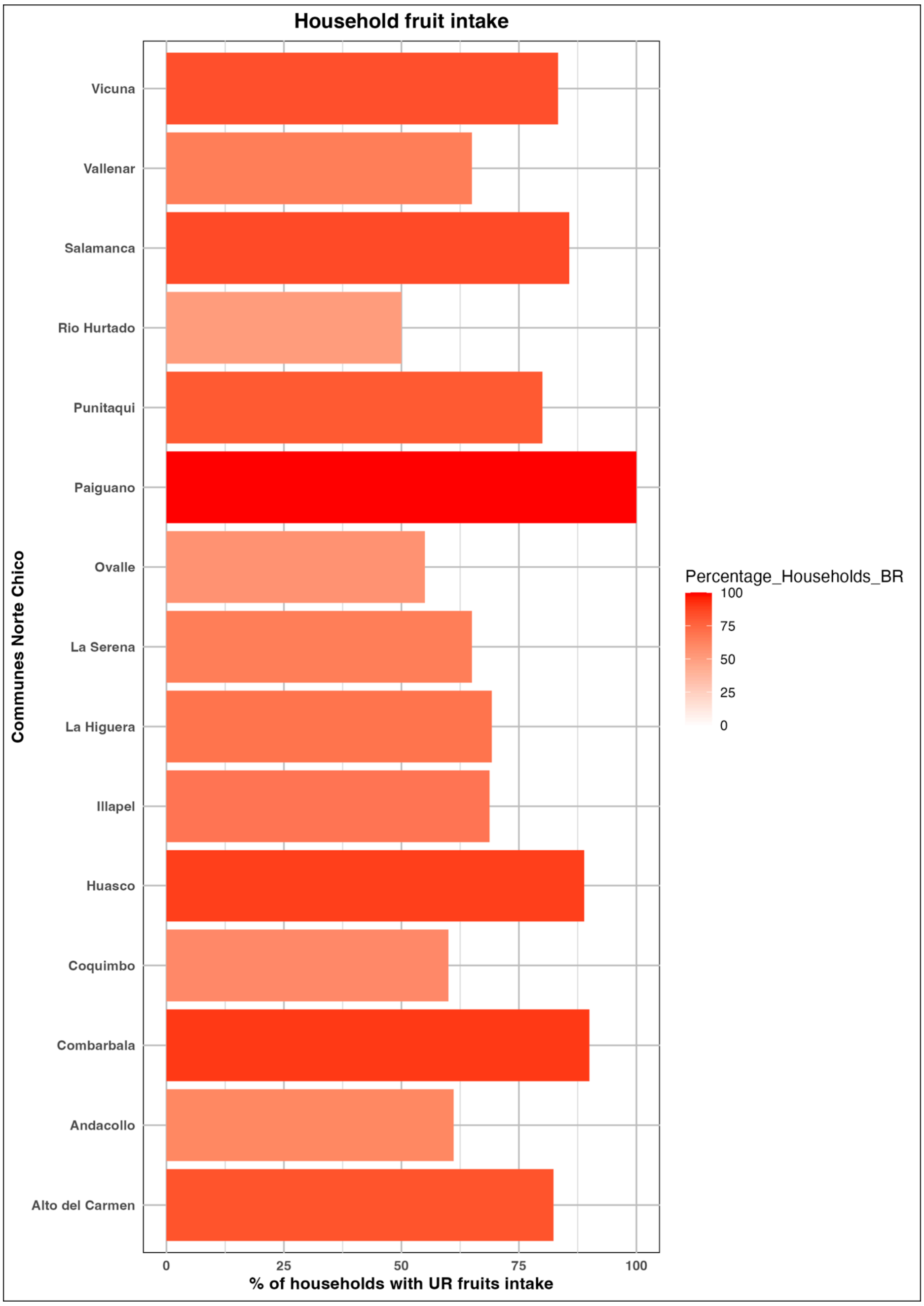

c. Zona Central

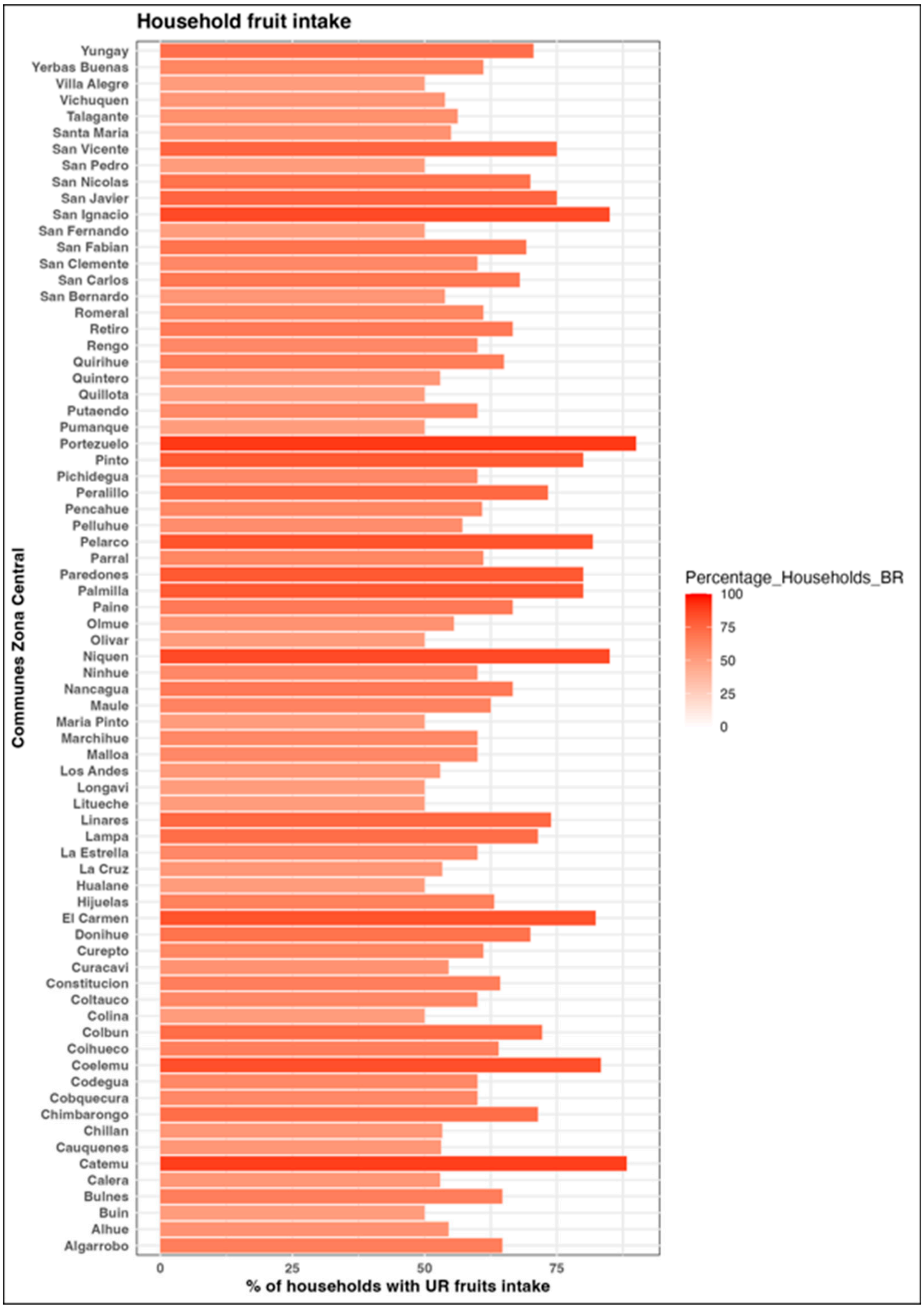
Household fruit intake
Communes Zona Central
Yungay
Yerbas Buenas
Villa Alegre
Vichuquen
Talagante
Santa Maria
San Vicente
San Pedro
San Nicolas
San Javier
San Ignacio
San Fernando
San Fabian
San Clemente
San Carlos
San Bernardo
Romeral
Retiro
Rengo
Quirihue
Quintero
Quillota
Putaendo
Pumanque
Portezuelo
Pinto
Pichidegua
Peralillo
Pencahue
Pelluhue
Pelarco
Parral
Paredones
Palmilla
Paine
Olmue
Olivar
Niquen
Ninhue
Nancagua
Maule
Maria Pinto
Marchihue
Malloa
Los Andes
Longavi
Litueche
Linares
Lampa
La Estrella
La Cruz
Hualane
Hijuelas
El Carmen
Donihue
Curepto
Curacavi
Constitucion
Coltauco
Colina
Colbun
Coihueco
Coelemu
Codegua
Cobquecura
Chimbarongo
Chillan
Cauquenes
Catemu
Calera
Bulnes
Buin
Alhue
Algarrobo
0
25
50
75
% of households with UR fruits intake
Percentage_Households_BR
100
75
50
25
0

d. Zona Sur

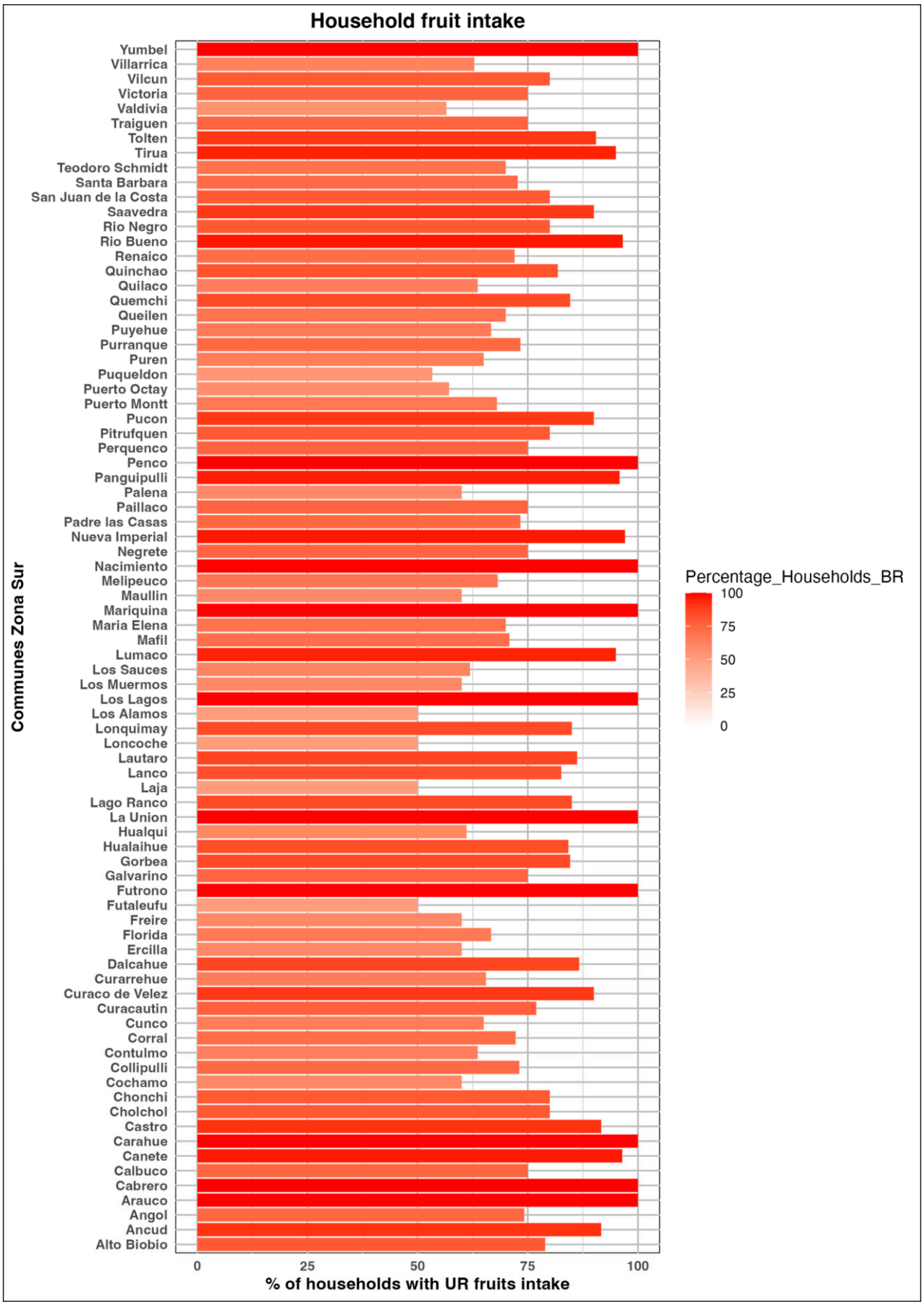

e. Zona Austral

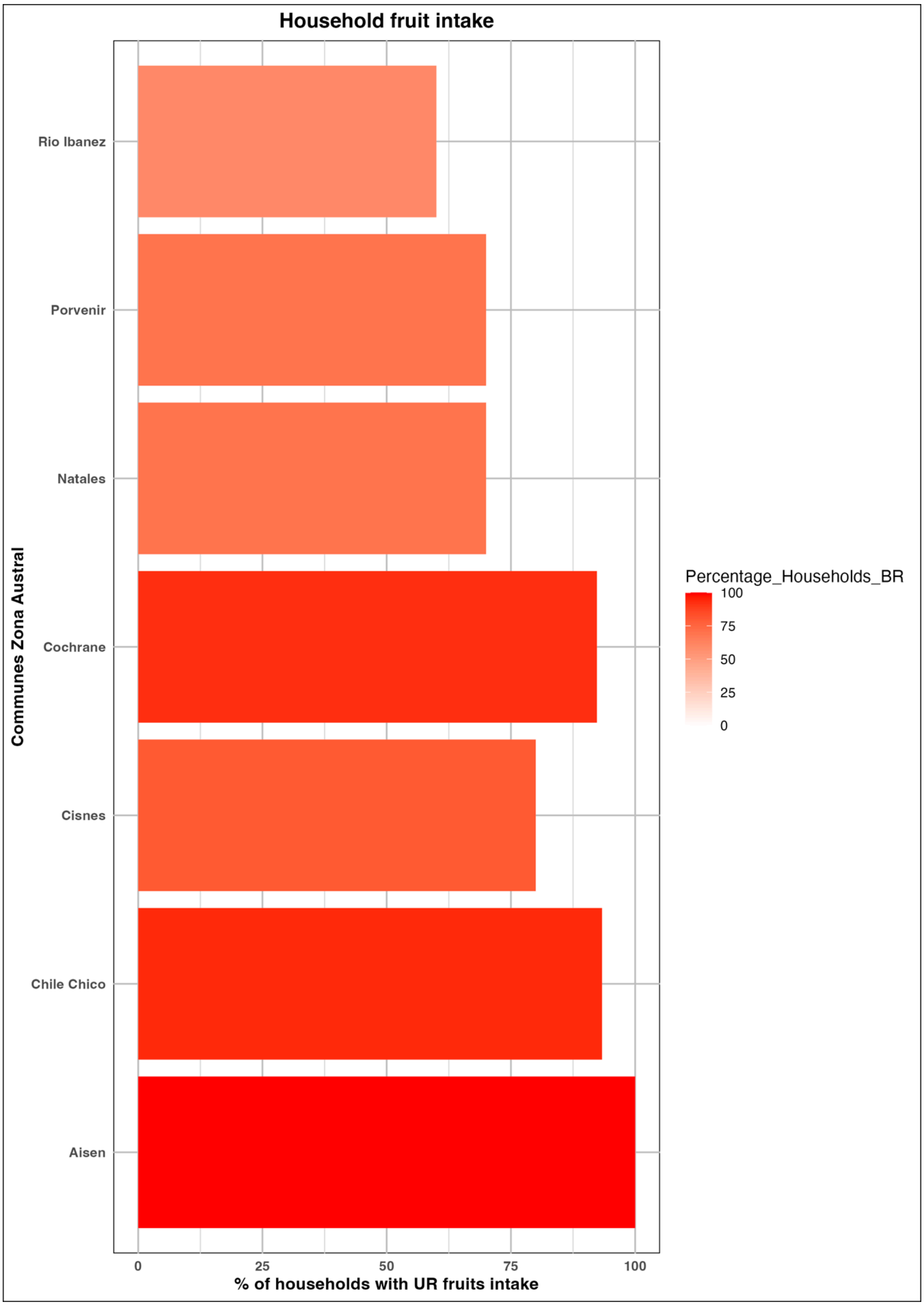

2. Figure S2. Vegetables
   a. Norte Grande

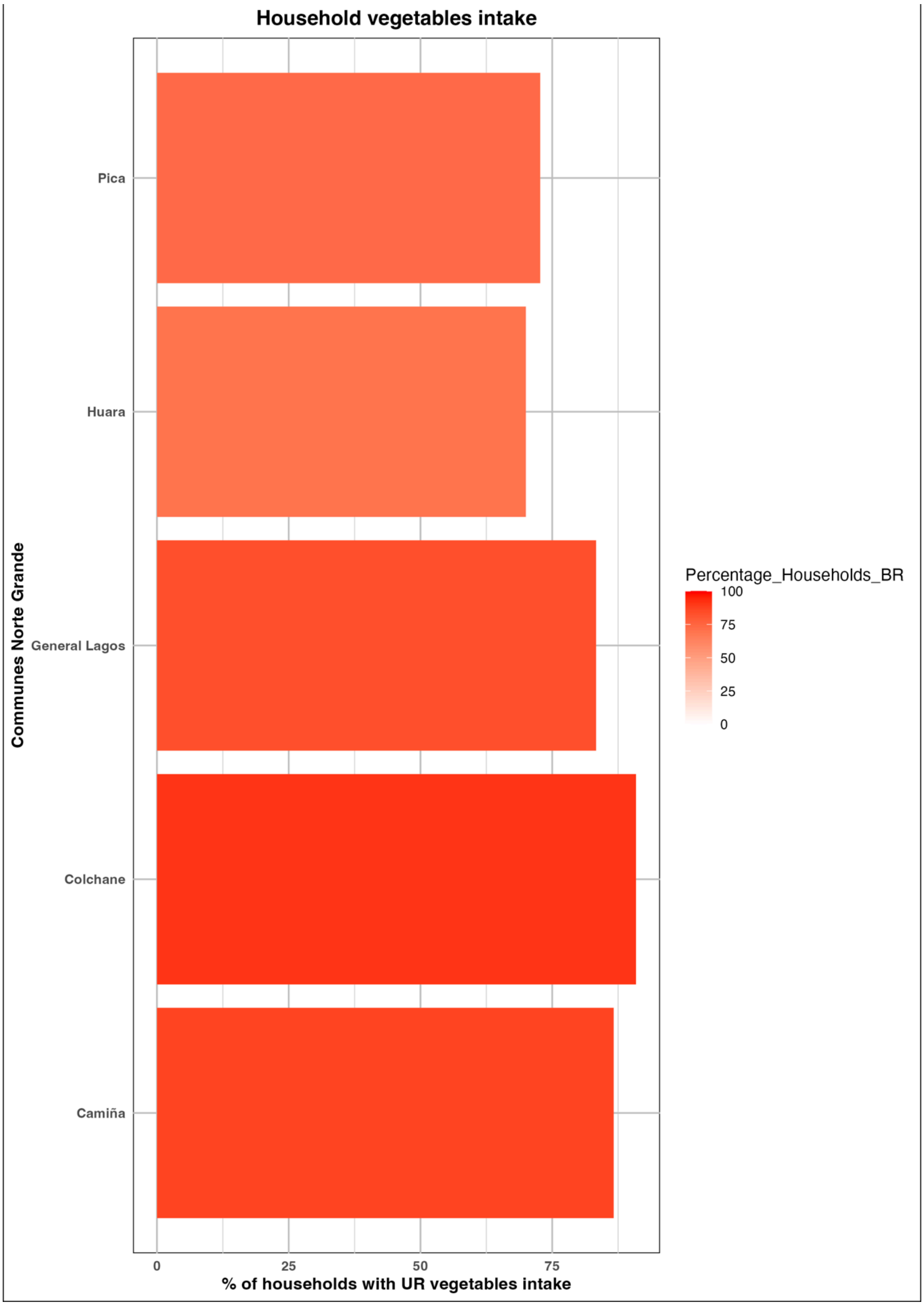

b. Norte Chico

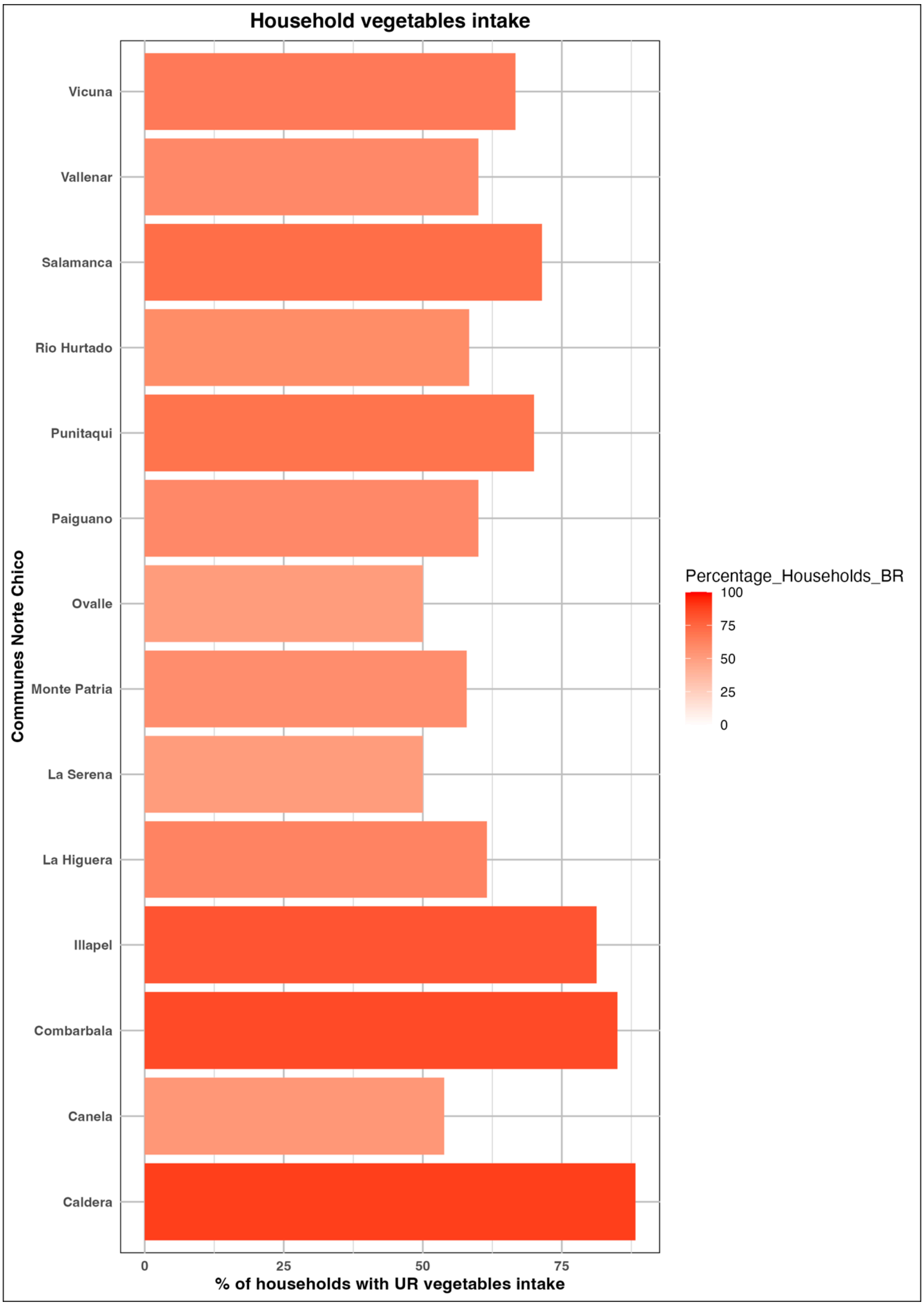

c. Zona Central

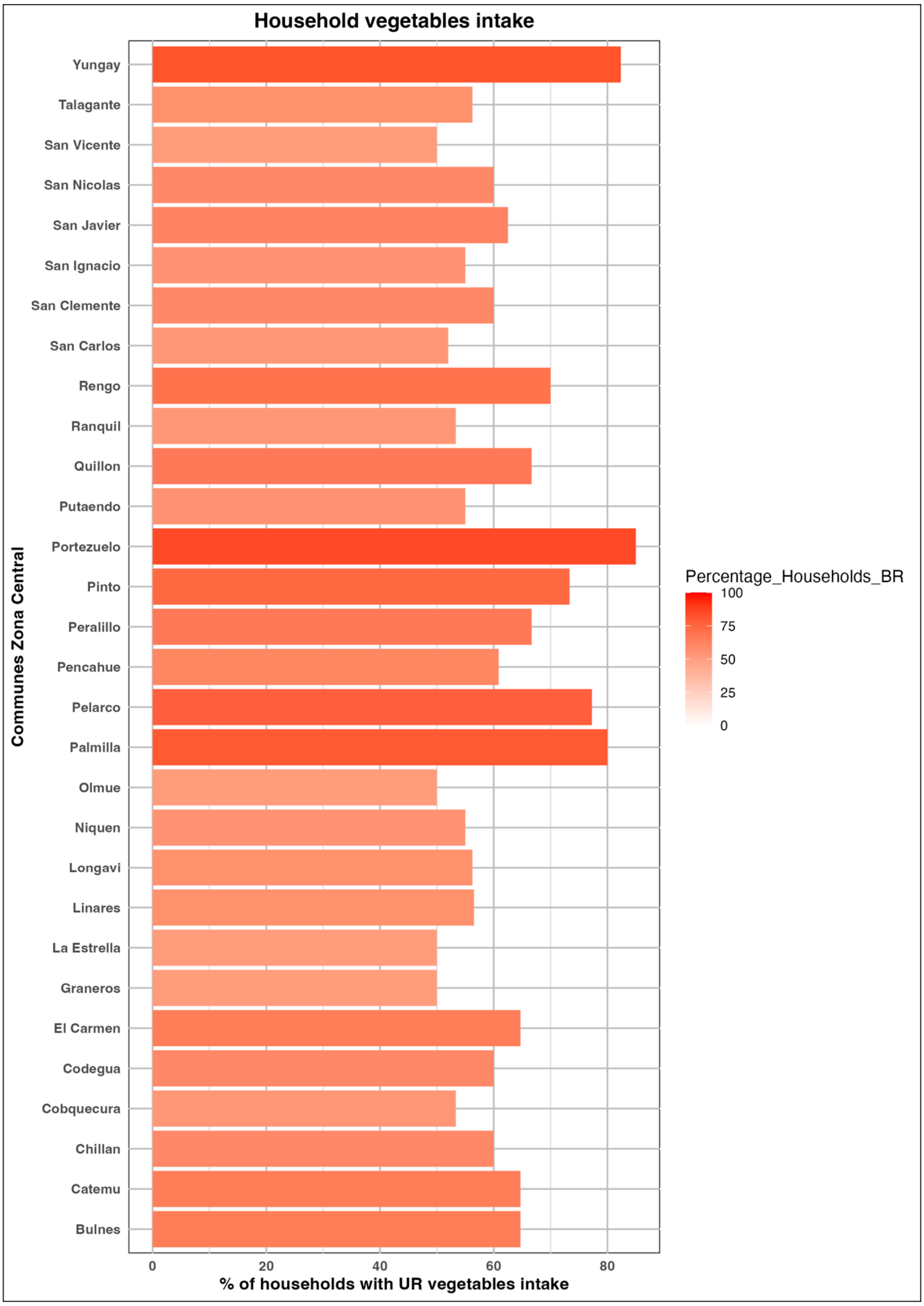
Household vegetables intake
Communes Zona Central
Yungay
Talagante
San Vicente
San Nicolas
San Javier
San Ignacio
San Clemente
San Carlos
Rengo
Ranquil
Quillon
Putaendo
Portezuelo
Pinto
Peralillo
Pencahue
Pelarco
Palmilla
Olmue
Niquen
Longavi
Linares
La Estrella
Graneros
El Carmen
Codegua
Cobquecura
Chillan
Catemu
Bulnes
0
20
40
60
80
% of households with UR vegetables intake
Percentage_Households_BR
100
75
50
25
0

d. Zona Sur

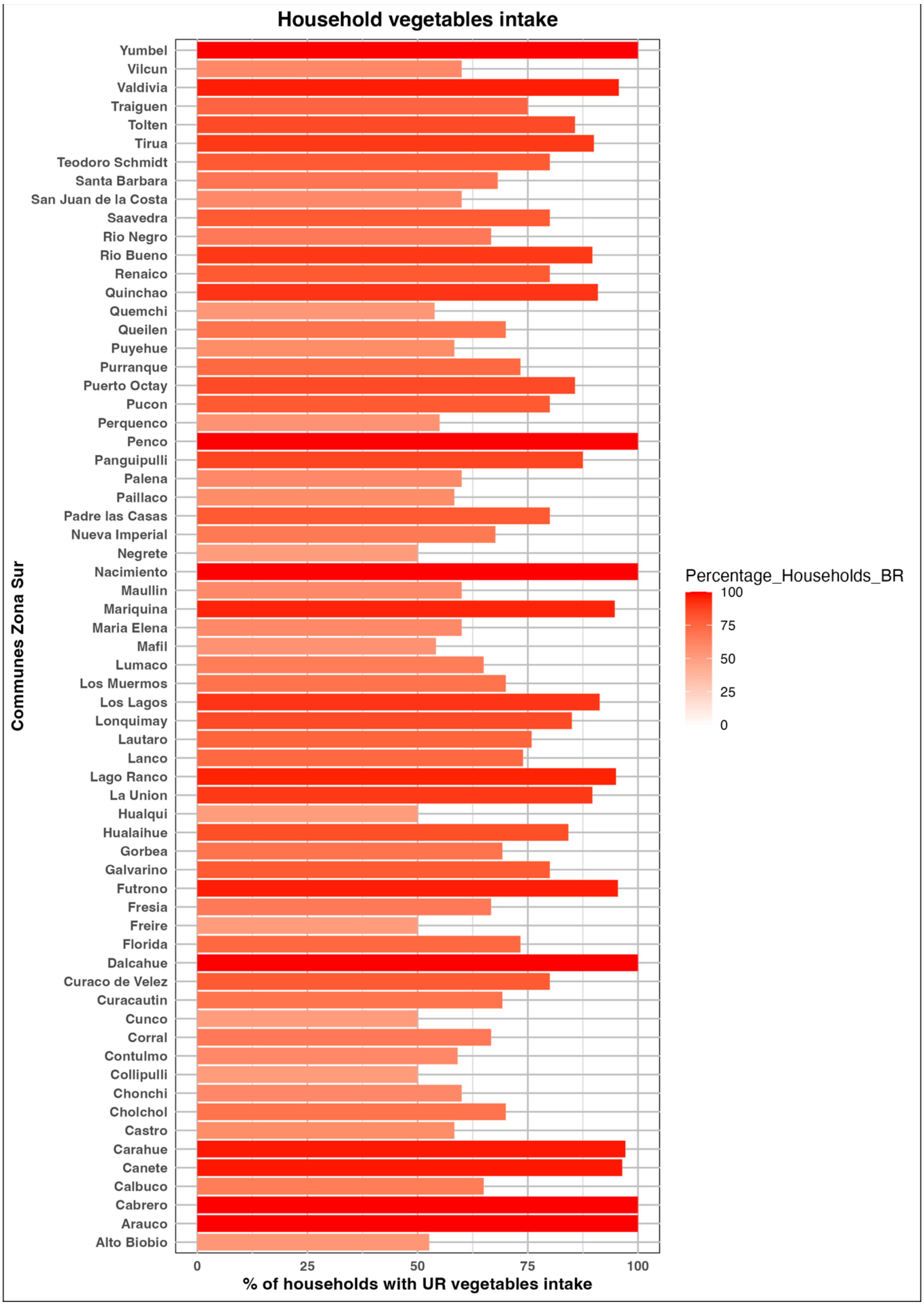

e. Zona Austral

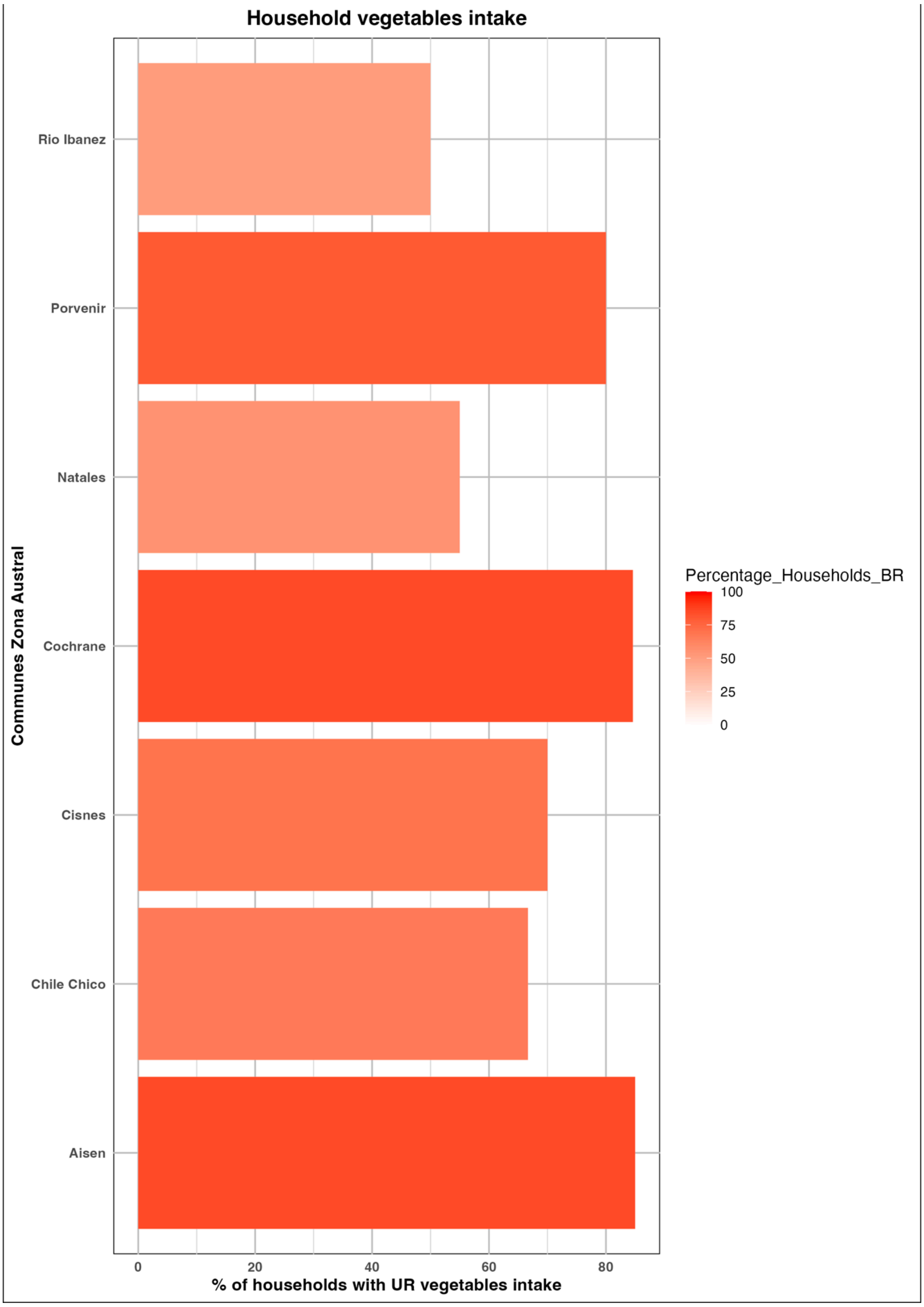

3. Figure S3. Dairy
   a. Norte Grande

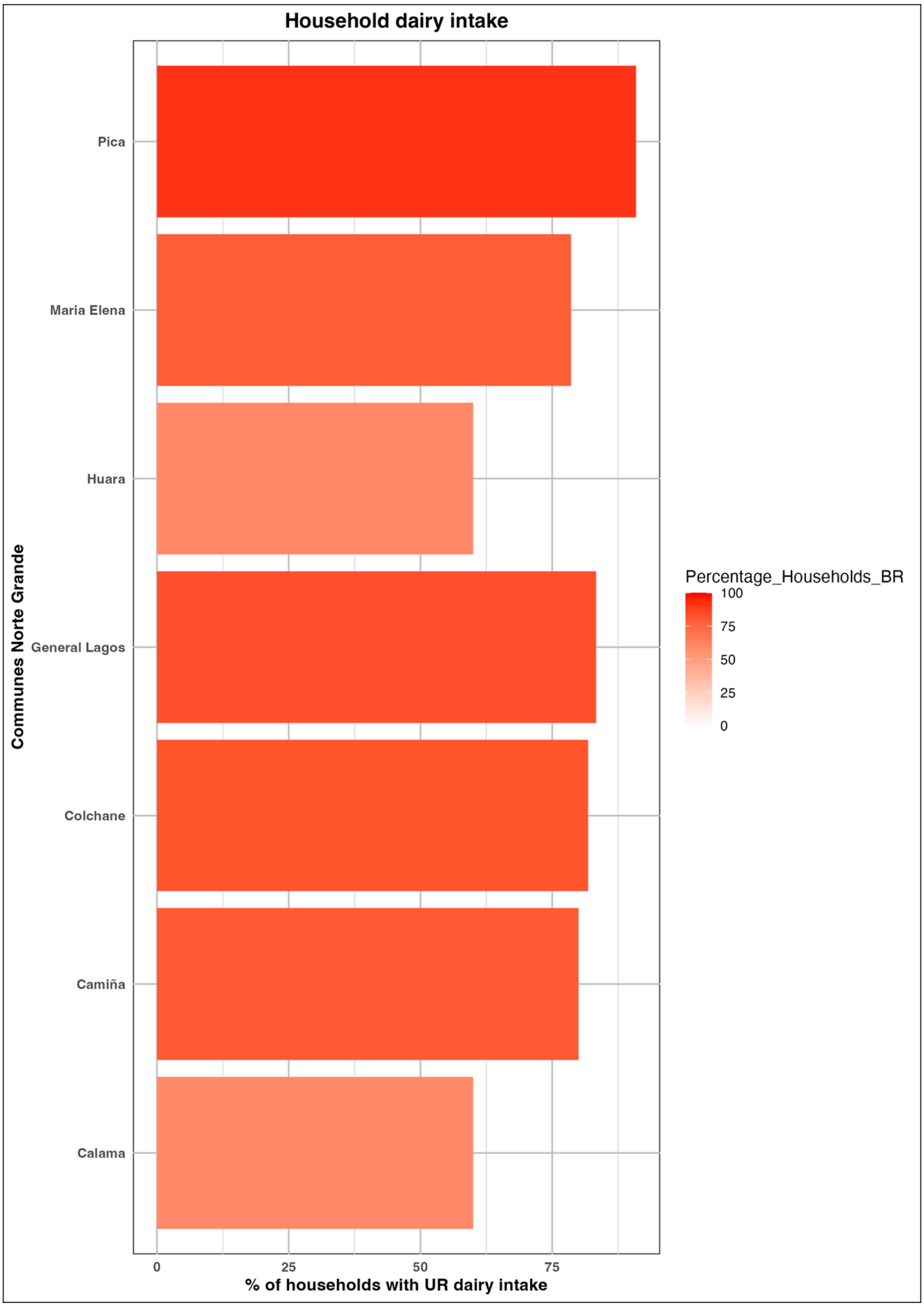

b. Norte Chico

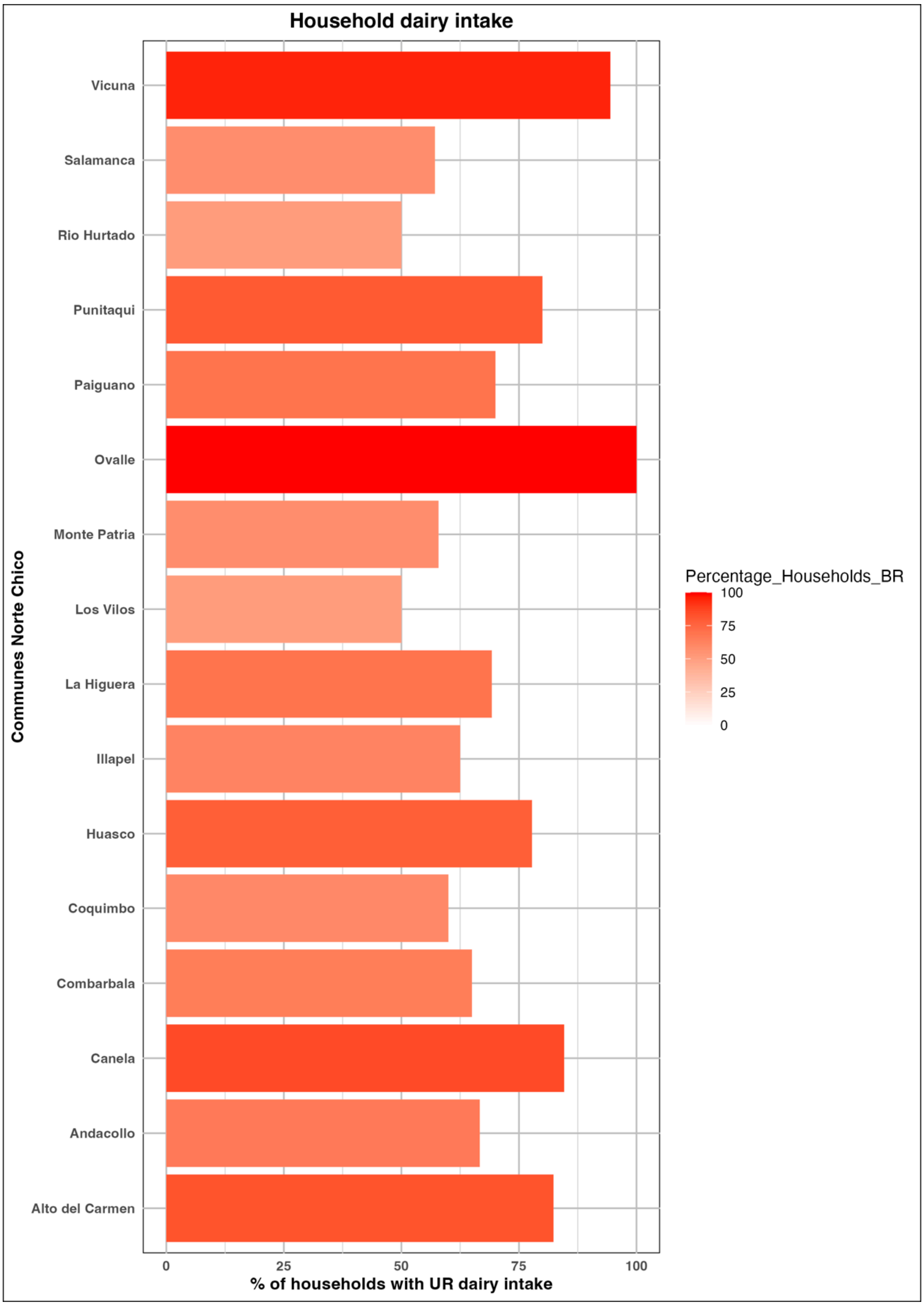

c. Zona Central

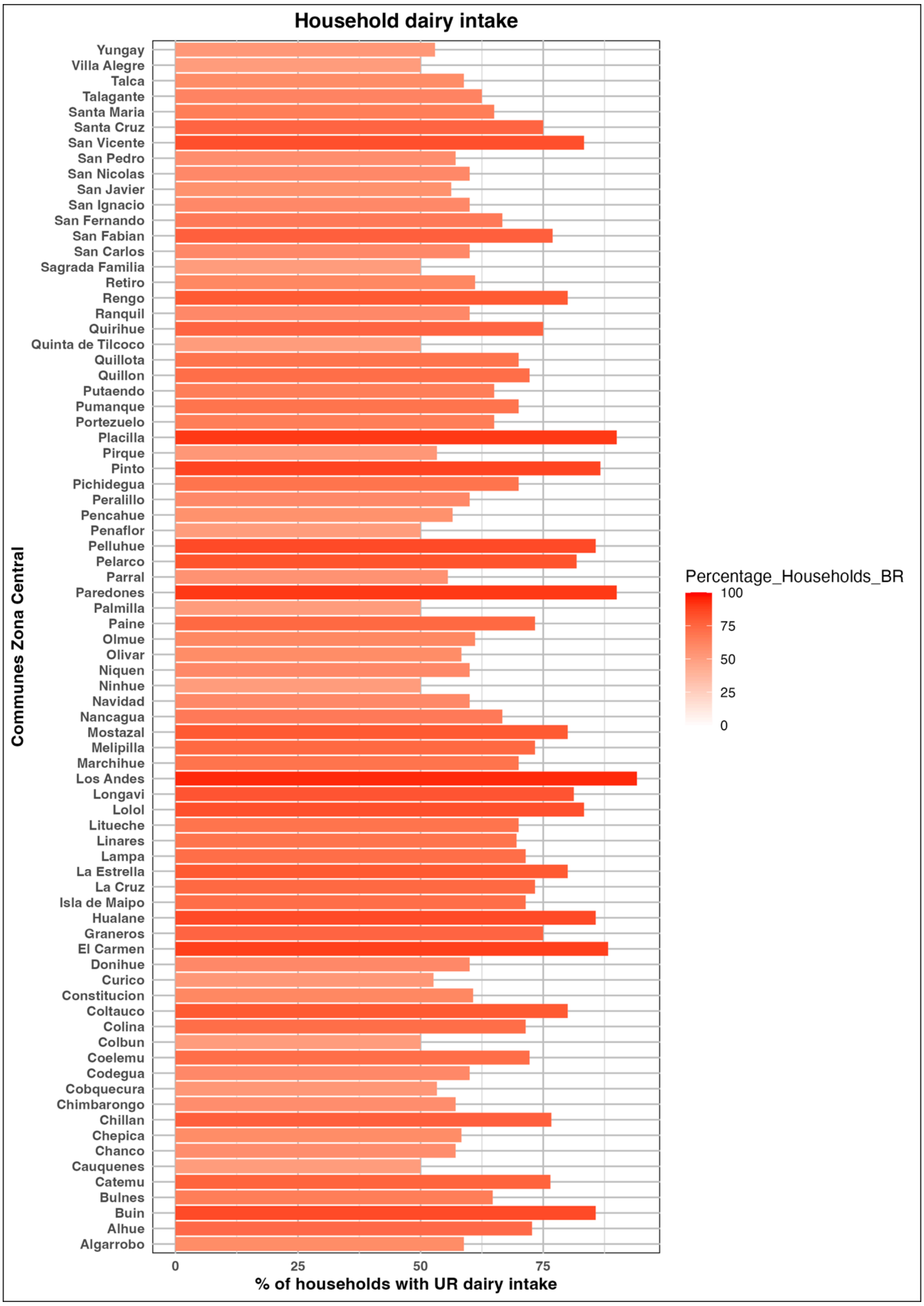
Household dairy intake
Communes Zona Central
Yungay
Villa Alegre
Talca
Talagante
Santa Maria
Santa Cruz
San Vicente
San Pedro
San Nicolas
San Javier
San Ignacio
San Fernando
San Fabian
San Carlos
Sagrada Familia
Retiro
Rengo
Ranquil
Quirihue
Quinta de Tilcoco
Quillota
Quillon
Putaendo
Pumanque
Portezuelo
Placilla
Pirque
Pinto
Pichidegua
Peralillo
Pencahue
Penaflor
Pelluhue
Pelarco
Parral
Paredones
Palmilla
Paine
Olmue
Olivar
Niquen
Ninhue
Navidad
Nancagua
Mostazal
Melipilla
Marchihue
Los Andes
Longavi
Lolol
Litueche
Linares
Lampa
La Estrella
La Cruz
Isla de Maipo
Hualane
Graneros
El Carmen
Donihue
Curico
Constitucion
Coltauco
Colina
Colbun
Coelemu
Codegua
Cobquecura
Chimbarongo
Chillan
Chepica
Chanco
Cauquenes
Catemu
Bulnes
Buin
Alhue
Algarrobo
0
25
50
75
% of households with UR dairy intake
Percentage_Households_BR
100
75
50
25
0

d. Zona Sur

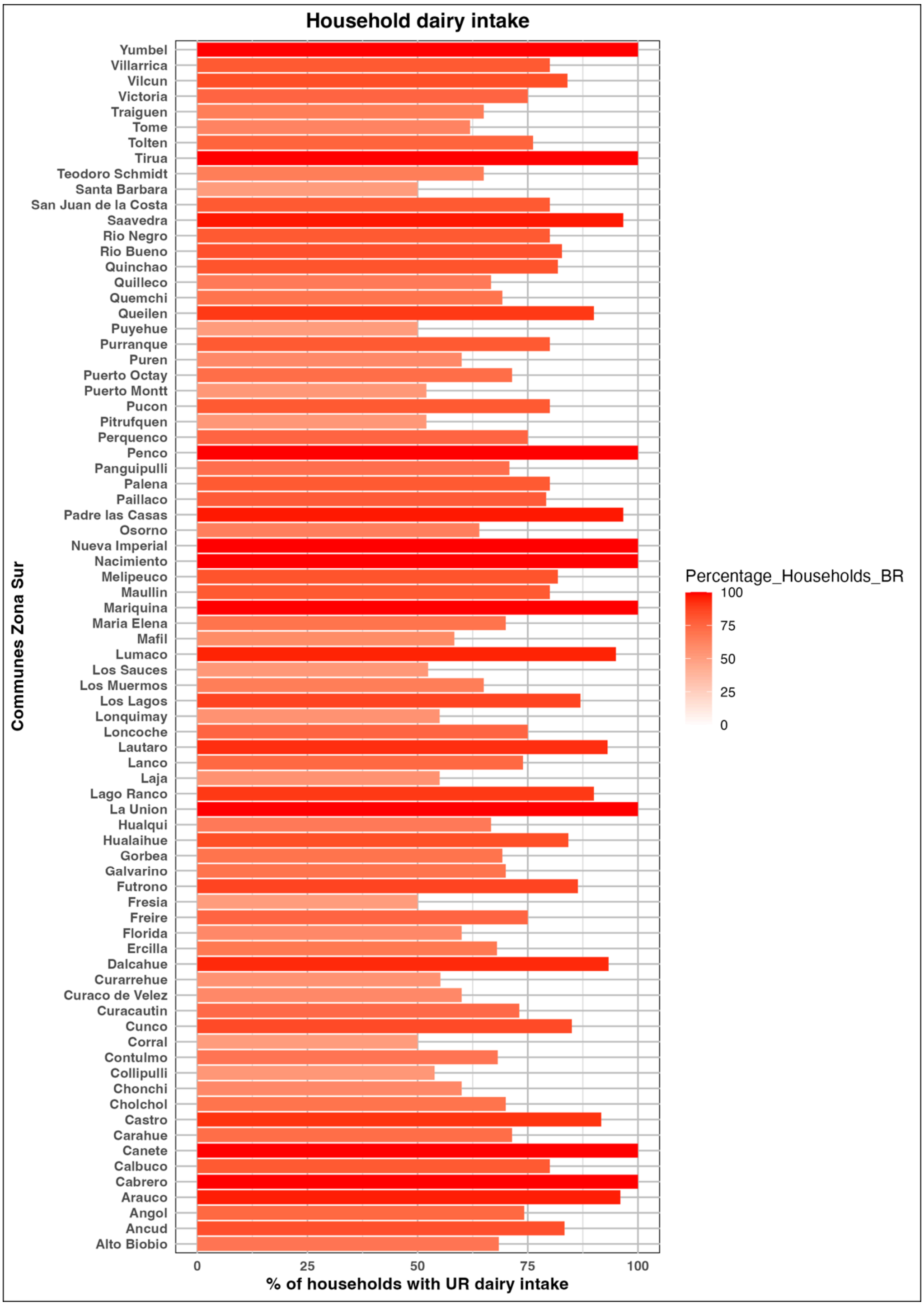

e. Zona Austral

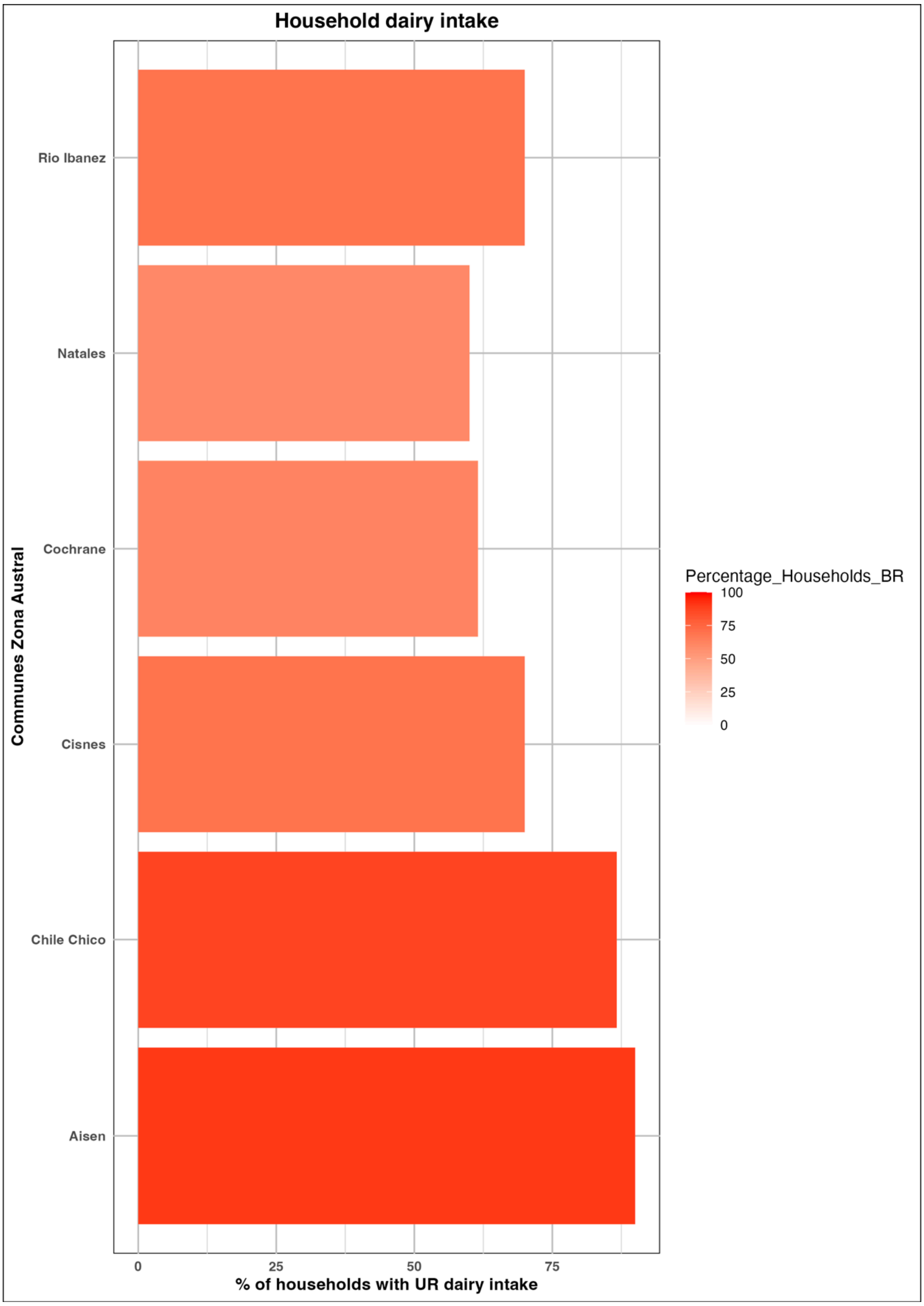

4. Figure S4. Fat
    a. Norte Grande

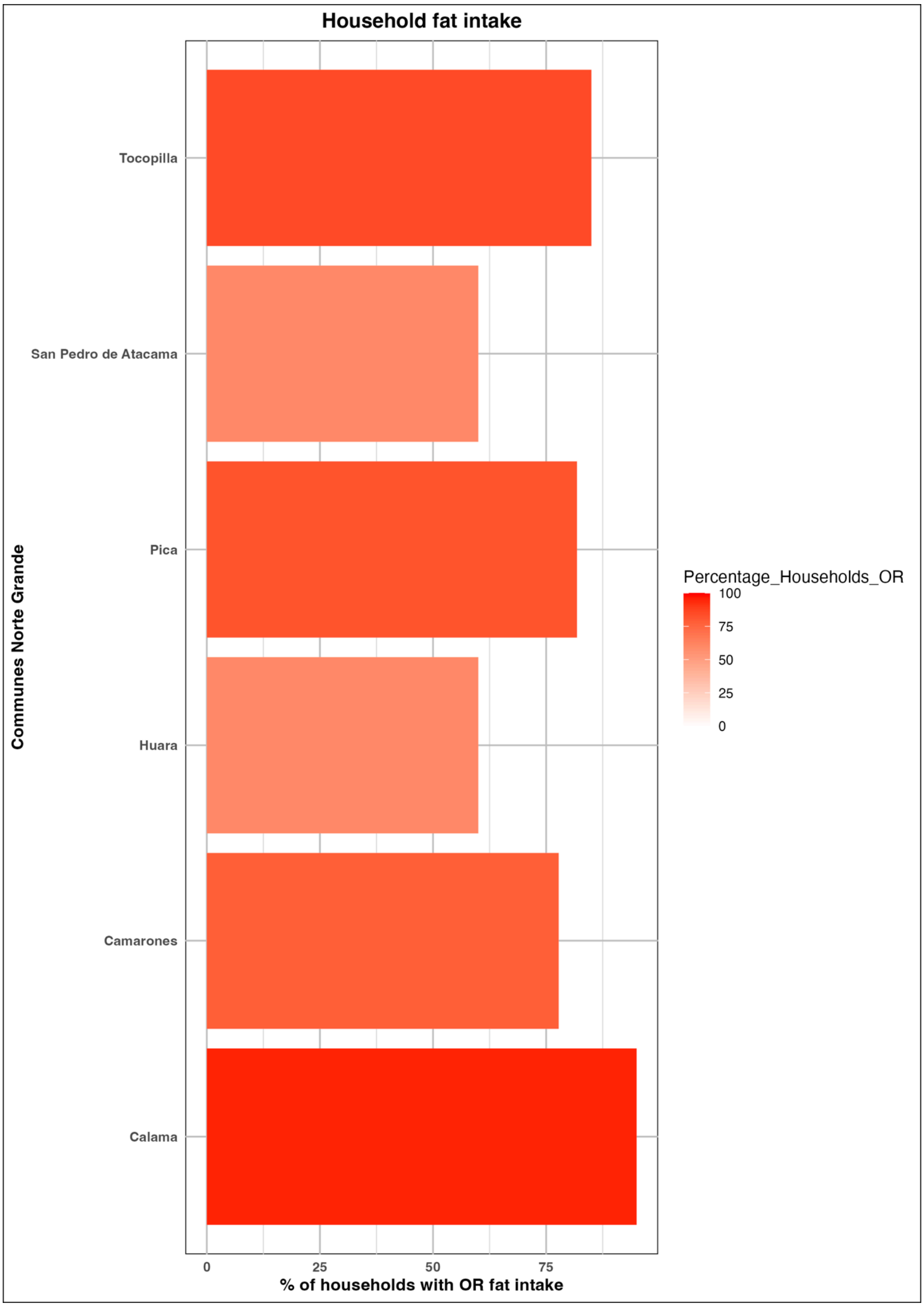

b. Norte Chico

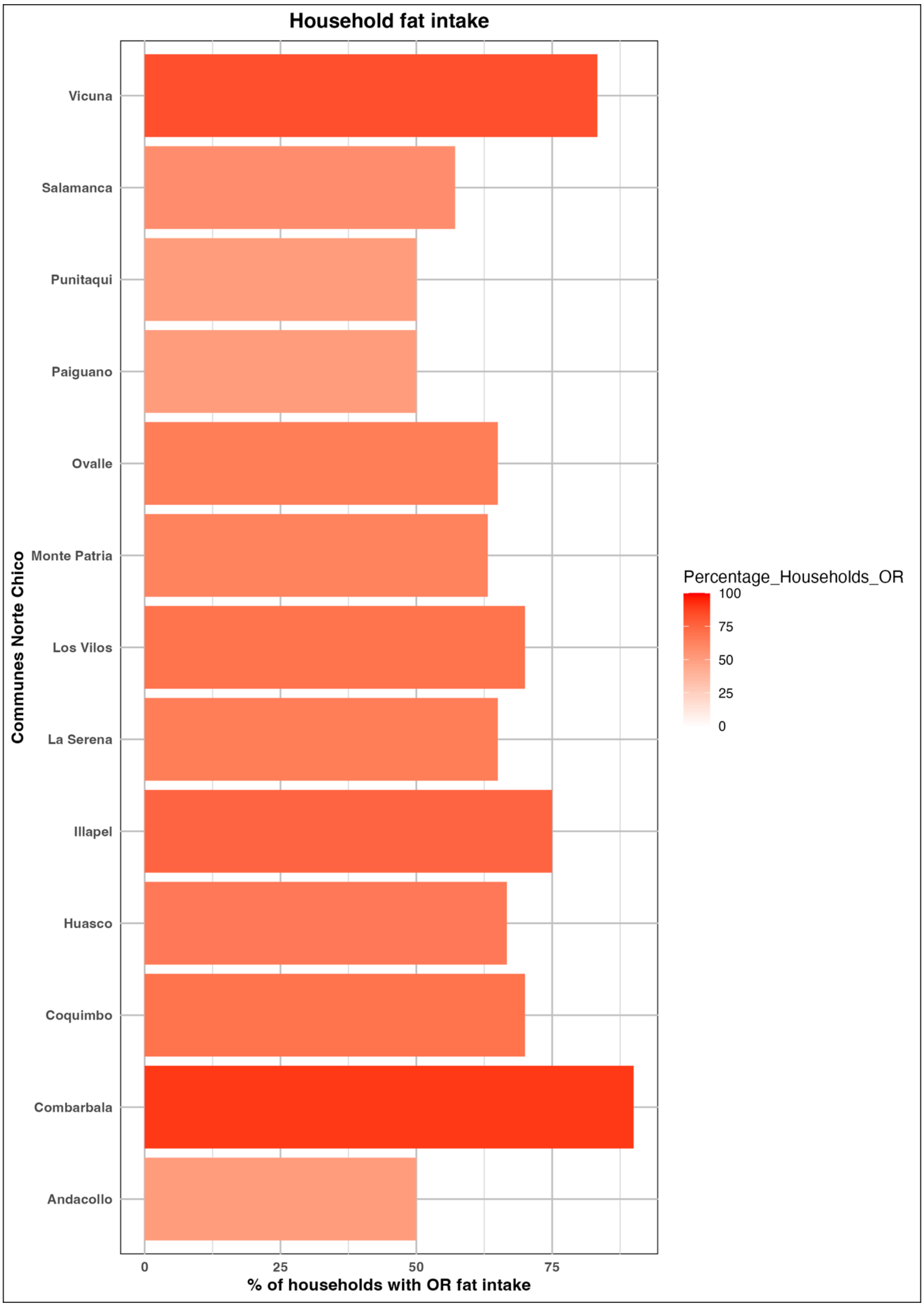

c. Zona Central

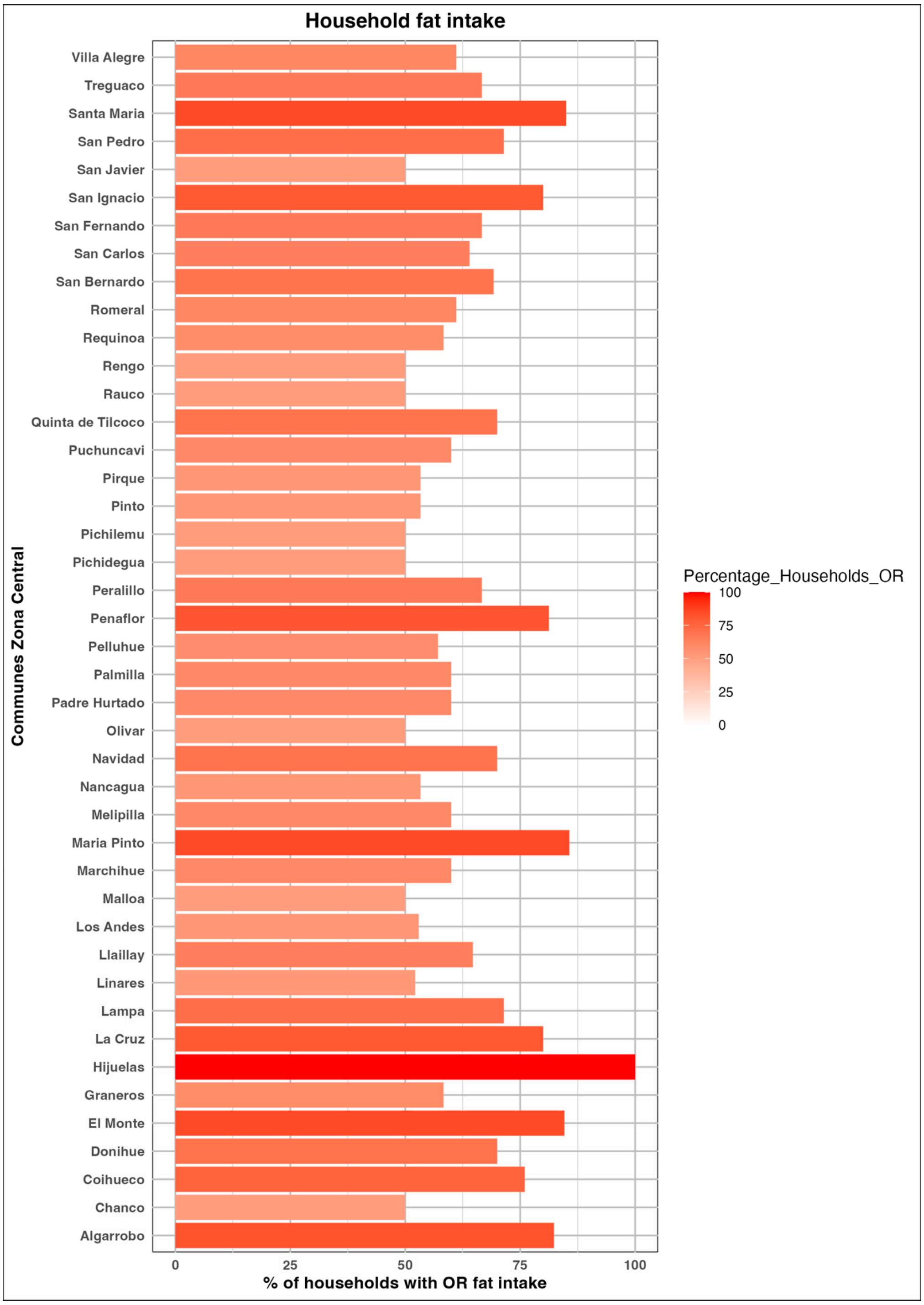
Household fat intake
Villa Alegre
Treguaco
Santa Maria
San Pedro
San Javier
San Ignacio
San Fernando
San Carlos
San Bernardo
Romeral
Requinoa
Rengo
Rauco
Quinta de Tilcoco
Puchuncavi
Pirque
Pinto
Pichilemu
Pichidegua
Peralillo
Penaflor
Pelluhue
Palmilla
Padre Hurtado
Olivar
Navidad
Nancagua
Melipilla
Maria Pinto
Marchihue
Malloa
Los Andes
Llaillay
Linares
Lampa
La Cruz
Hijuelas
Graneros
El Monte
Donihue
Coihueco
Chanco
Algarrobo
Communes Zona Central
0
25
50
75
100
% of households with OR fat intake
Percentage_Households_OR
100
75
50
25
0

d. Zona Sur

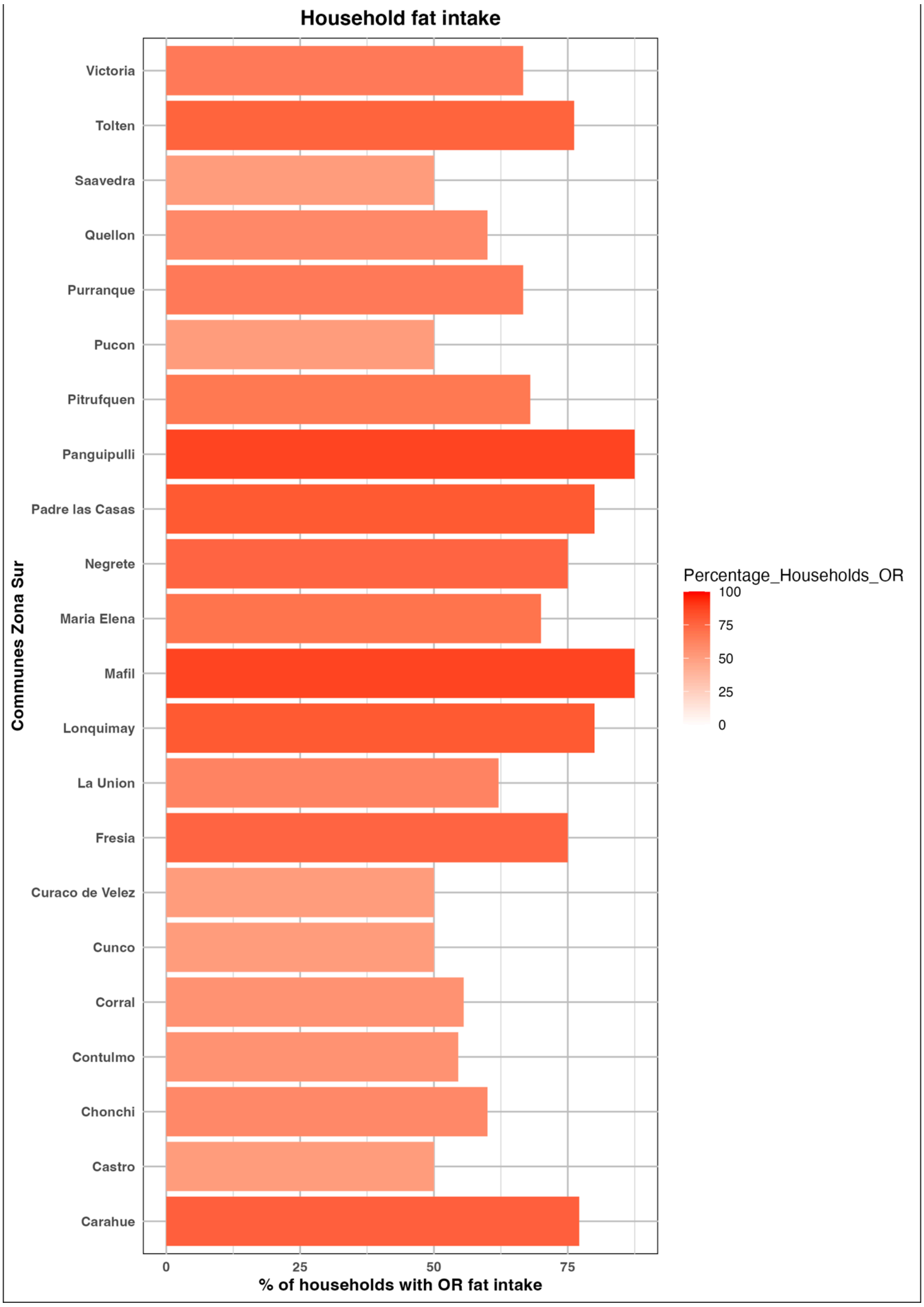

e. Zona Austral

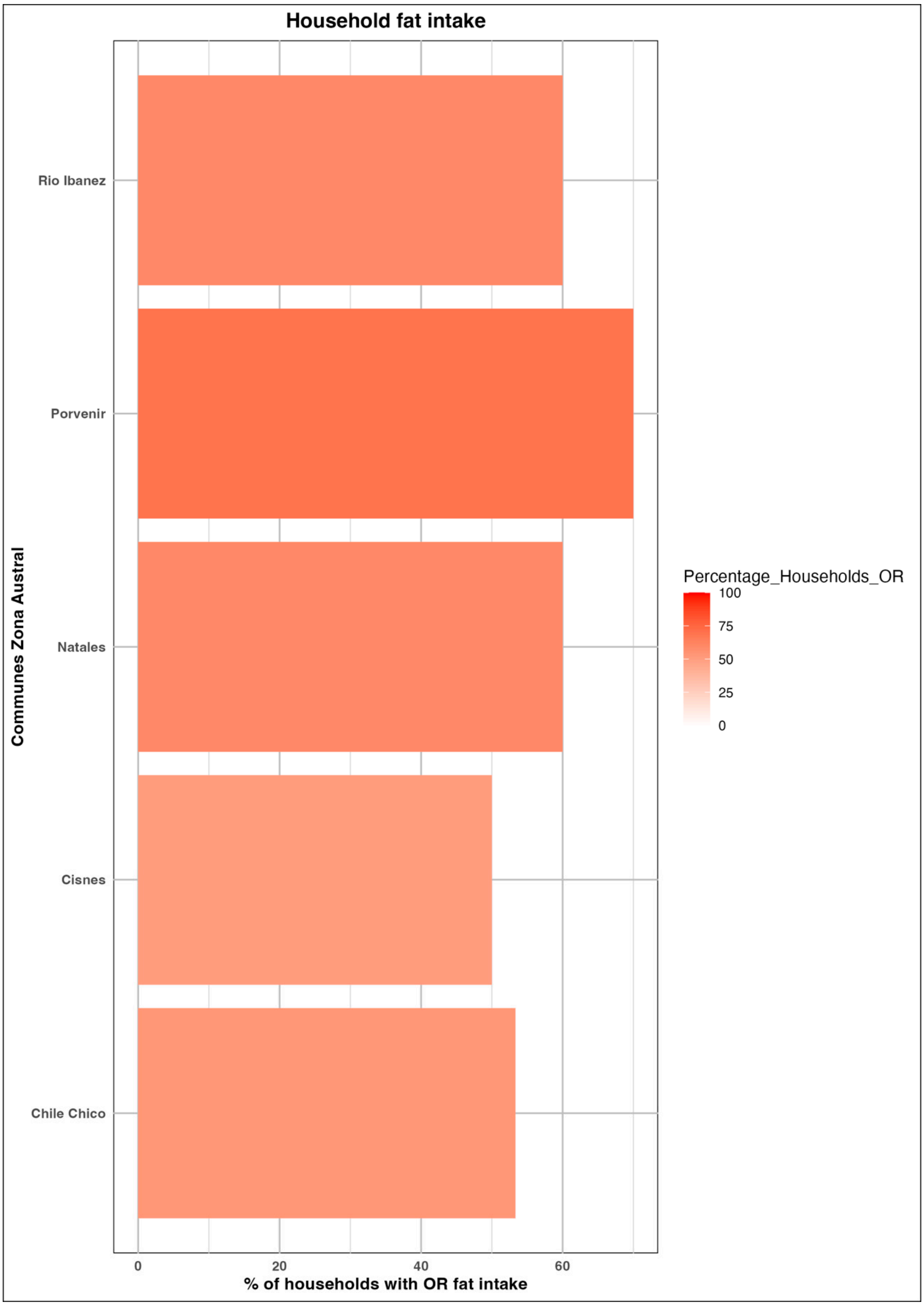

Household fat intake
Rio Ibanez
Porvenir
Natales
Cisnes
Chile Chico
Communes Zona Austral
0
20
40
60
% of households with OR fat intake
Percentage_Households_OR
100
75
50
25
0

5. Figure S5. Sugar

a. Norte Grande

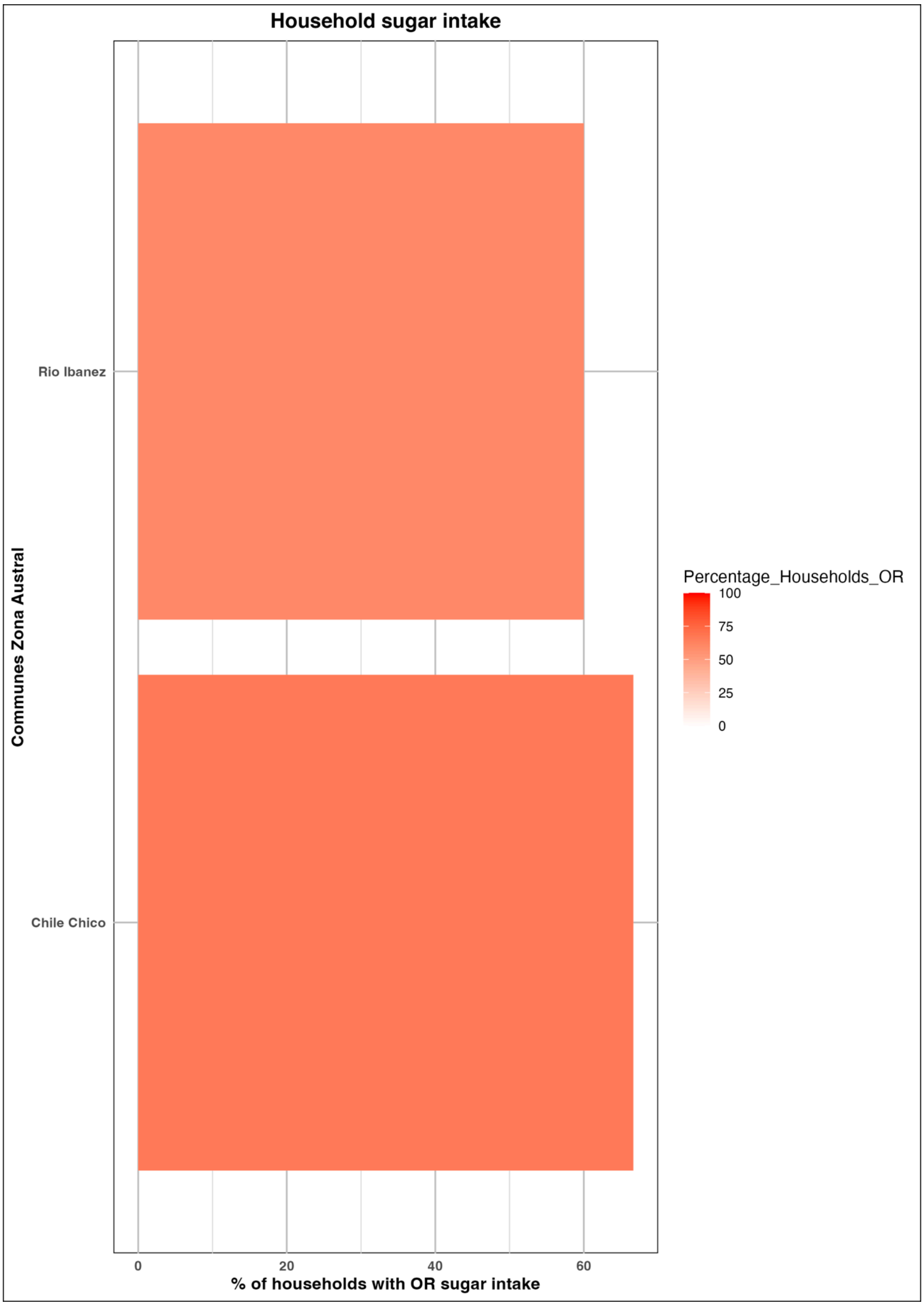

b. Norte Chico

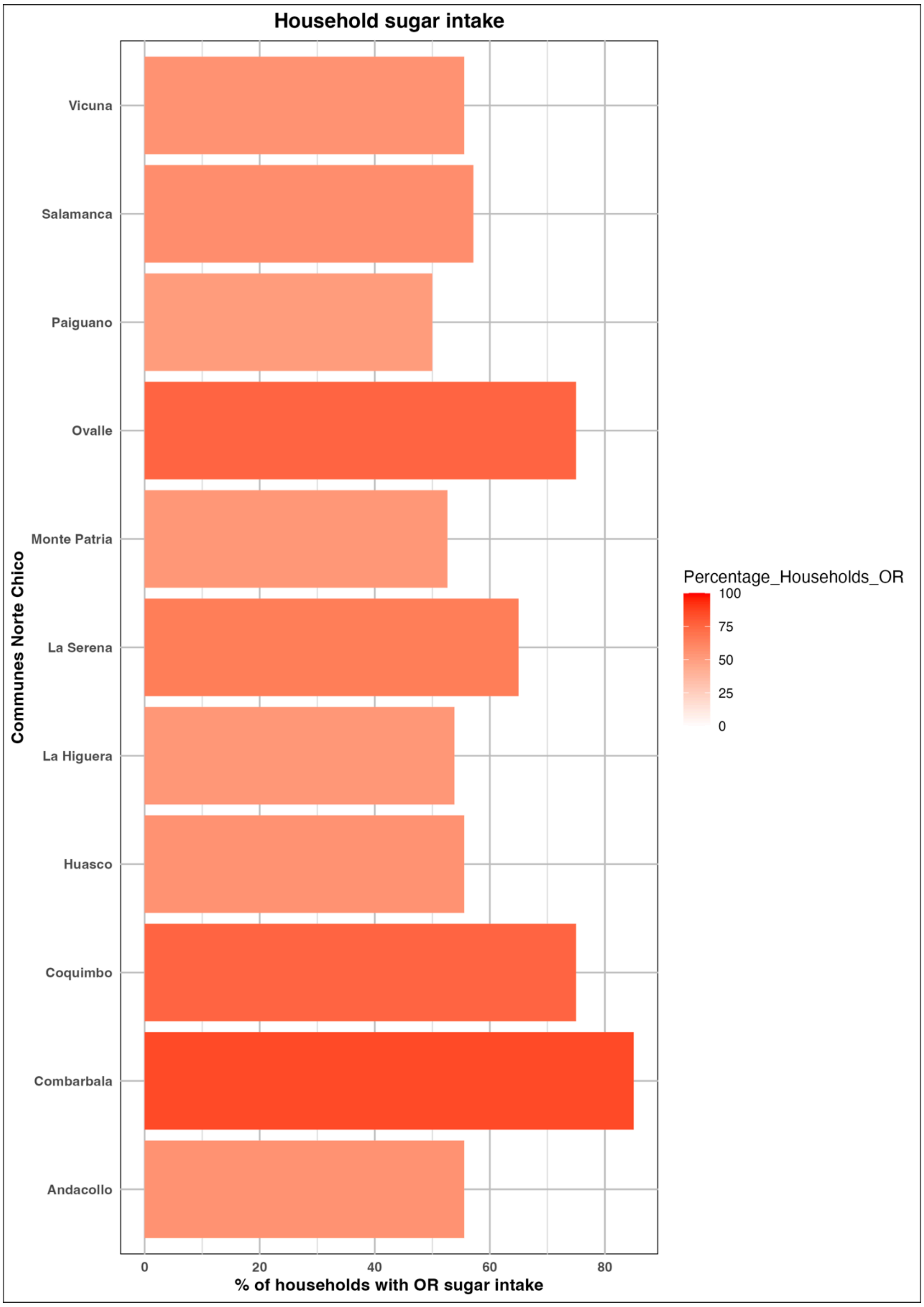

c. Zona Central

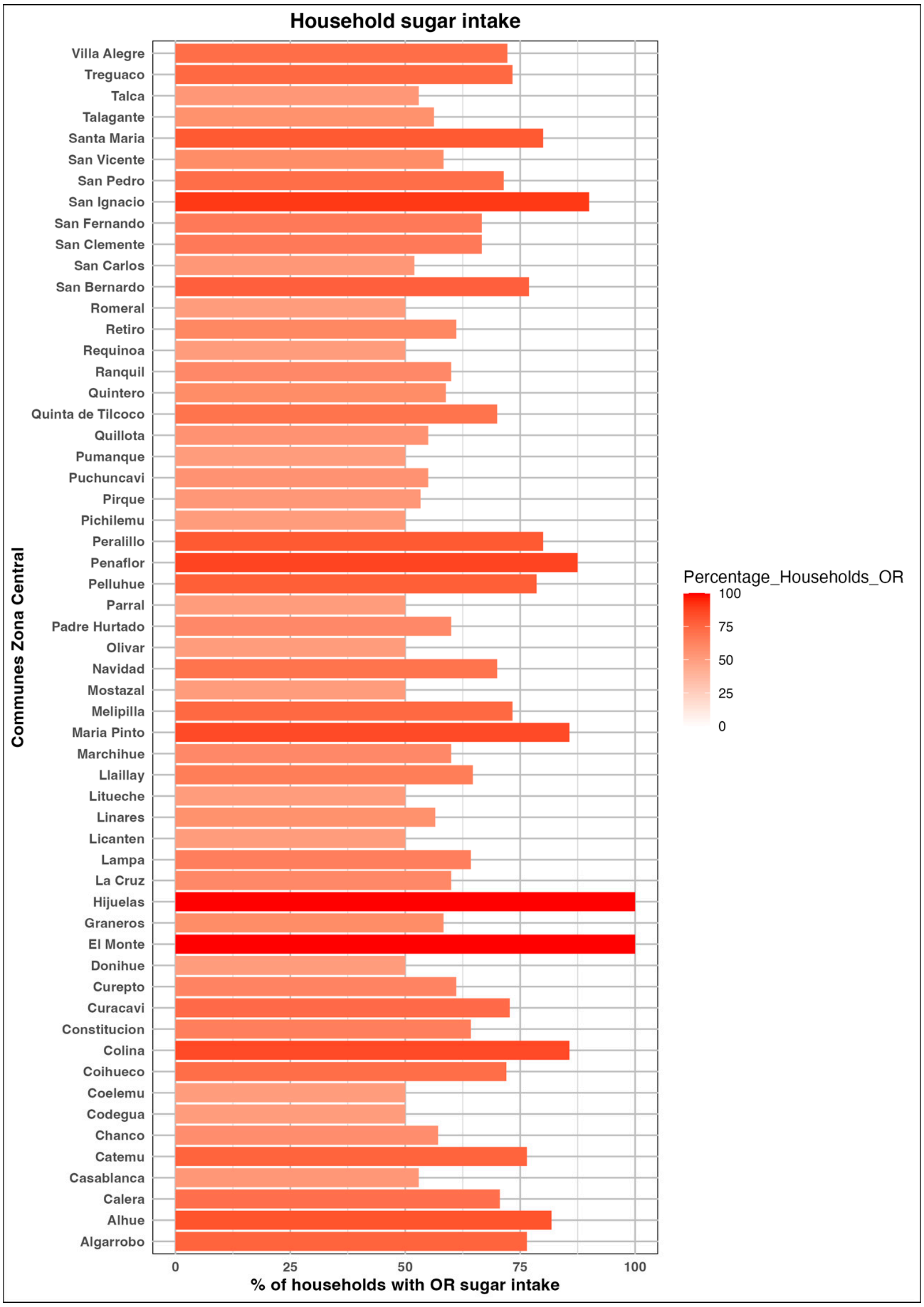

Household sugar intake
Communes Zona Central
Villa Alegre
Treguaco
Talca
Talagante
Santa Maria
San Vicente
San Pedro
San Ignacio
San Fernando
San Clemente
San Carlos
San Bernardo
Romeral
Retiro
Requinoa
Ranquil
Quintero
Quinta de Tilcoco
Quillota
Pumanque
Puchuncavi
Pirque
Pichilemu
Peralillo
Penaflor
Pelluhue
Parral
Padre Hurtado
Olivar
Navidad
Mostazal
Melipilla
Maria Pinto
Marchihue
Llaillay
Litueche
Linares
Licanten
Lampa
La Cruz
Hijuelas
Graneros
El Monte
Donihue
Curepto
Curacavi
Constitucion
Colina
Coihueco
Coelemu
Codegua
Chanco
Catemu
Casablanca
Calera
Alhue
Algarrobo
0
25
50
75
100
% of households with OR sugar intake
Percentage_Households_OR
100
75
50
25
0

d. Zona Sur

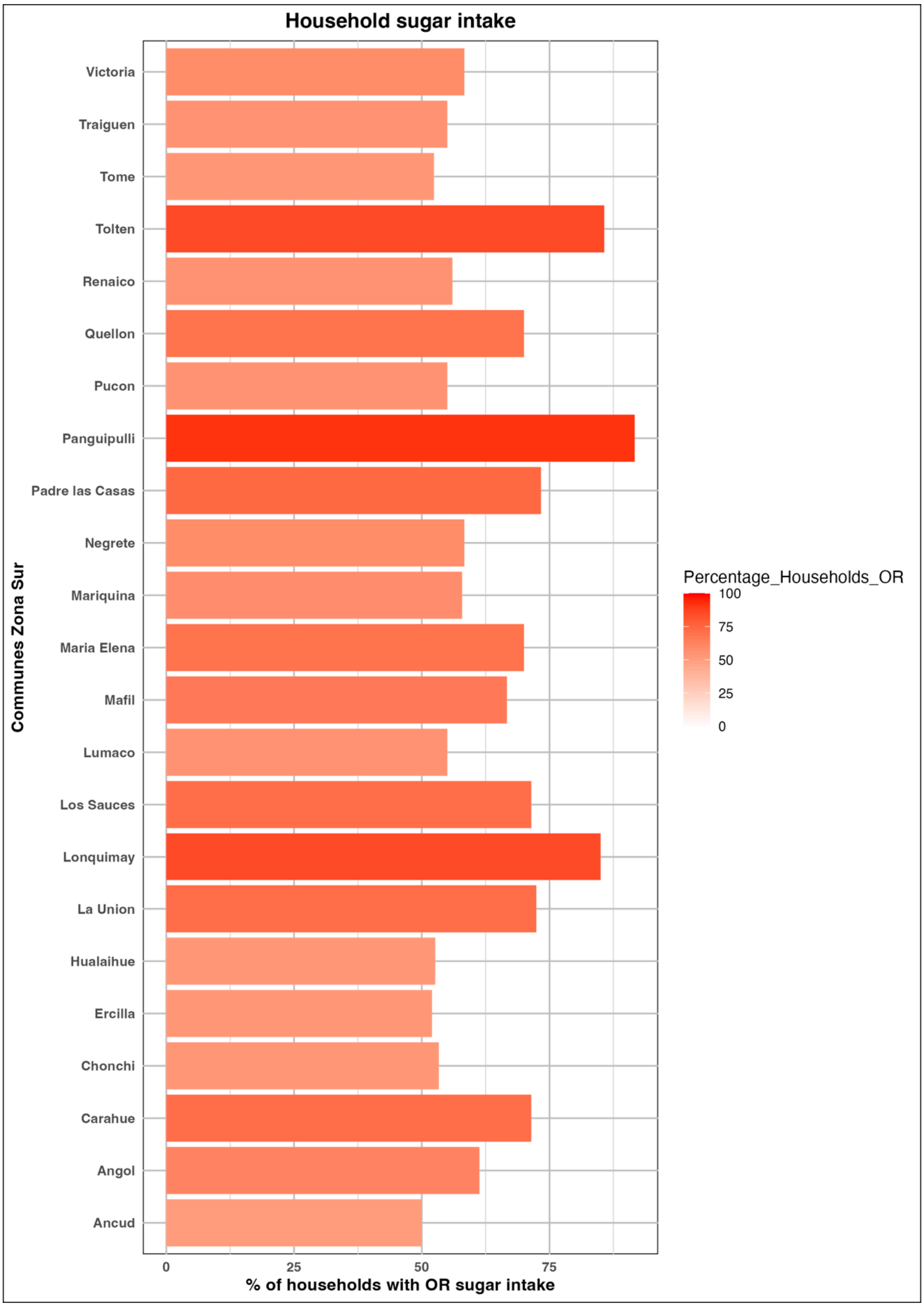

e. Zona Austral

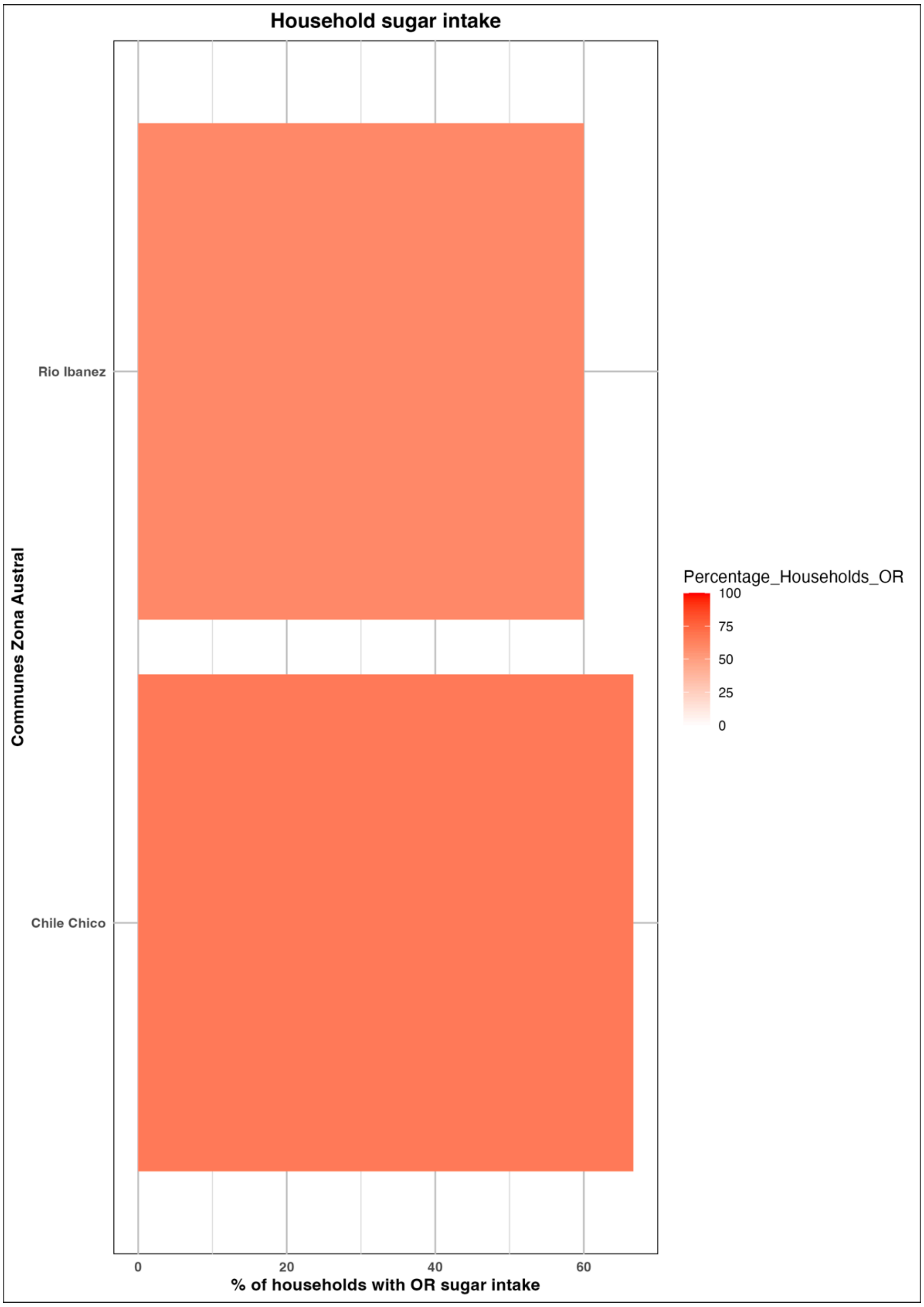
Household sugar intake
Rio Ibanez
Chile Chico
Communes Zona Austral
0
20
40
60
% of households with OR sugar intake
Percentage_Households_OR
100
75
50
25
0
